# Functional promiscuity in a mammalian chemosensory system: extensive expression of vomeronasal receptors in the main olfactory epithelium of mouse lemurs

**DOI:** 10.3389/fnana.2014.00102

**Published:** 2014-09-24

**Authors:** Philipp Hohenbrink, Silke Dempewolf, Elke Zimmermann, Nicholas I. Mundy, Ute Radespiel

**Affiliations:** ^1^Institute of Zoology, University of Veterinary Medicine HannoverHannover, Germany; ^2^Department of Zoology, University of CambridgeCambridge, UK

**Keywords:** MOE, VNO, *V1R*, *V2R*, *TRPC2*, olfaction, primate, *Microcebus*

## Abstract

The vomeronasal organ (VNO) is functional in most terrestrial mammals, though progressively reduced in the primate lineage, and is used for intraspecific communication and predator recognition. Vomeronasal receptor (VR) genes comprise two families of chemosensory genes (*V1R* and *V2R*) that have been considered to be specific for the VNO. However, recently a large number of VRs were reported to be expressed in the main olfactory epithelium (MOE) of mice, but there is little knowledge of the expression of these genes outside of rodents. To explore the function of VR genes in mammalian evolution, we analyzed and compared the expression of 64 *V1R* and 2 *V2R* genes in the VNO and the MOE of the gray mouse lemur (*Microcebus murinus*), the primate with the largest known VR repertoire. We furthermore compared expression patterns in adults of both sexes and seasons, and in an infant. A large proportion (83–97%) of the VR loci was expressed in the VNO of all individuals. The repertoire in the infant was as rich as in adults, indicating reliance on olfactory communication from early postnatal development onwards. In concordance with mice, we also detected extensive expression of VRs in the MOE, with proportions of expressed loci in individuals ranging from 29 to 45%. *TRPC2*, which encodes a channel protein crucial for signal transduction via VRs, was co-expressed in the MOE in all individuals indicating likely functionality of expressed VR genes in the MOE. In summary, the large VR repertoire in mouse lemurs seems to be highly functional. Given the differences in the neural pathways of MOE and VNO signals, which project to higher cortical brain centers or the limbic system, respectively, this raises the intriguing possibility that the evolution of MOE-expression of VRs enabled mouse lemurs to adaptively diversify the processing of VR-encoded olfactory information.

## Introduction

Most terrestrial mammals use two olfactory systems, the main olfactory system based on the main olfactory epithelium (MOE) and the accessory olfactory system based on the vomeronasal organ (VNO). The MOE has traditionally been considered to detect small odorants and a few pheromones (Restrepo et al., 2004), whereas the VNO is specialized for the detection of pheromones, signature mixtures and kairomones (e.g., predator cues) (Keverne, 1999; Isogai et al., 2011) and is therefore essential for intraspecific communication and predator avoidance. Two types of vomeronasal receptors (VRs) are described which have been thought to be primarily expressed in the VNO based on studies in rodents: vomeronasal 1 receptors (*V1R*s, Dulac and Axel, 1995) and vomeronasal 2 receptors (*V2R*s, Herrada and Dulac, 1997; Matsunami and Buck, 1997; Ryba and Tirindelli, 1997). The two receptor types seem to be specialized for ligands of different size: *V1R*s bind smaller molecules (Leinders-Zufall et al., 2000), whereas *V2R*s bind larger peptides (like Major Histocompatibility Complex: Leinders-Zufall et al., 2004; Endocrine gland-Secreting Peptide 1: Kimoto et al., 2005; and Major Urinary Proteins: Chamero et al., 2007). The broader pattern of expression is yet unclear and a few cells of the MOE in mice and goats express single *V1R*s (Wakabayashi et al., 2002; Karunadasa et al., 2006; Ohara et al., 2009). However, a recent study using nano Cap Analysis of Gene Expression revealed the expression of a large proportion of VRs in the mouse MOE (112 of 191 tested *V1R*s and 96 of 123 *V2R*s; Pascarella et al., 2014). Both types of VRs use the cation channel *TRPC2* (Transient Receptor Potential channel type C2) in the signal transduction pathway (Liman et al., 1999). Although one study did not find *TRPC2* expression outside of the mouse VNO (Zhang et al., 2010), another study reported a very faint signal for *TRPC2* in the MOE on Northern blots and a faint signal in a small population of MOE cells by *in situ* hybridization (Liman et al., 1999). Furthermore, tag clusters associated to *TRPC2* were found in MOE tissue of mice (Pascarella et al., 2014).

In primates it was observed that the size of the VNO correlates with its functionality: it is well developed and functional in strepsirrhine primates (lemurs and lorisoids), smaller but still functional in tarsiers and most New World monkeys and vestigial or absent in catarrhine primates (Old World monkeys, apes and humans) (Martin, 1990; Smith et al., 2011; Garrett et al., 2013). *V1R* repertoires in strepsirrhine primates (78–214 estimated genes) are as large as in rodents (89–239 estimated genes, Young et al., 2010), whereas only 2 intact *V2R* genes have been described in strepsirrhines (Hohenbrink et al., 2013) in contrast to about 100 intact genes in rodents (Young and Trask, 2007). Catarrhine primates lack functional *V2R*s and the few intact *V1R*s are not considered to be functional in the VNO, since *TRPC2* is a pseudogene in catarrhines including humans (Liman and Innan, 2003; Zhang and Webb, 2003; Young and Trask, 2007; Young et al., 2010).

Strepsirrhine primates are ideal to study the vomeronasal system in non-model species as they heavily rely on olfactory communication (Jolly, 1966; Perret, 1995; Buesching et al., 1998; Braune et al., 2005; DelBarco-Trillo et al., 2011) and olfactory predator recognition has been described in mouse lemurs (Sündermann et al., 2008; Kappel et al., 2011). Although large genomic repertoires of vomeronasal receptors (VRs) have been identified in strepsirrhine primates (Young et al., 2010; Yoder et al., 2014), little is known about the expression patterns of the genes. Thus it is currently unclear whether the pattern of expression found in mice is representative of other mammals, which has important consequences for understanding the functional role of VRs.

To further our understanding of the role of VNO and MOE in the olfactory system of mammals and to shed light on the evolution of this sensory modality in primates, we analyze the expression patterns of VRs and *TRPC2* in gray mouse lemurs (*Microcebus murinus*). Among strepsirrhine primates mouse lemurs are an ideal model system as large parts of their genome is available and the organization of their *V1R* and *V2R* repertoires has already been analyzed (Young et al., 2010; Hohenbrink et al., 2012, 2013). Notably, they possess one of the largest predicted *V1R* repertoire of any mammal, comprising at least nine monophyletic gene clusters that have mostly evolved under positive selection (Hohenbrink et al., 2012). For this study we analyzed expression of a large proportion of *V1R* and *V2R* loci in the VNO and MOE of both sexes and both seasons (reproductive and non-reproductive season). We also studied a young infant to identify expression patterns at a very early postnatal developmental age. We ask whether a high proportion of the VR repertoire is expressed in the VNO, and if this varies with age, sex, or season. We also ask whether VR expression in the MOE is as widespread as recently shown in mice.

## Materials and methods

### Tissue collection

Complete VNOs and MOE tissues were collected from freshly deceased gray mouse lemurs that were euthanized for veterinary reasons or died naturally. No animal was sacrificed for the purpose of this study. All individuals were housed in the breeding colony of the Institute of Zoology of the University of Veterinary Medicine in Hannover under seasonal light regimes (see Wrogemann and Zimmermann, 2001 for details). The international and national guidelines for the care and housing of animals were followed namely the NRC Guide for the Care and Use of Laboratory Animals, the European Directive 2010/63/EU on the protection of animals used for scientific purposes, and the German Animal Welfare Act (licensed by the Bezirksregierung Hannover, reference number AZ 33.9-42502-05-10A080, and by Ordnungsamt, Gewerbe- und Veterinärabteilung, Landeshauptstadt Hannover, AZ 42500/1H). Animals used in this study died between March 2012 and April 2013 and were not visually impaired (Dubicanac, personal communication). We selected four different adults to detect seasonal and sex differences (see Table 1 for details). We also tested one female infant and removed tissue from the maxilloturbinals of one adult as a negative control. Maxilloturbinal tissue is non-sensory (no olfactory mucosa) but nasal tissue in close proximity to the vomeronasal organ (Smith and Rossie, 2008). If maxilloturbinal tissue was not contaminated with VNO cells during its removal, we assume that MOE tissue was also not contaminated with VNO cells and potential expression patterns of VRs in MOE tissue shown in this study relate to actual expression in the MOE. We were not able to obtain a MOE sample for the female that died during the non-reproductive season due to technical problems during the dissection. All tissue samples were stored in RNAlater (Qiagen) at −80°C immediately after removal and RNA was extracted up to one year after storage using the RNeasy Micro Kit (Qiagen). The 260/280 nm absorbance ratios of all extracted RNA samples were close to 2.0 indicating high RNA purity (instead of 1.8 for DNA). Transcription into cDNA was performed with the Quantitect Reverse Transcription Kit (Qiagen) and N6 primer, according to manufacturer's instructions. The extraction and transcription kits both contain steps by the manufacturer to eliminate genomic DNA by DNase treatment. The successful extraction of RNA and synthesis of cDNA was confirmed by amplifying an intron-spanning segment of *ACTB* (beta-actin; ACTB-Exon-4-fw CTG TGC TGT CCC TGT ACG C, ACTB-Exon-6-rv AGT CCG CCT AGA AGC ATT TG), for which intronless cDNA was shorter on an agarose gel than genomic control DNA. Amplification and sequencing of an intron-spanning fragment of *TRPC2* was used to confirm its expression. Primers for *TRPC2* were TRPC2-A2 (TGA GCC AGG ACT ATG GCT TT) and TRPC2-B (Talarico, 2006: CAG GTT CCC ACA CCA GAT G), which bind to exon 3 and 4, respectively (PCR conditions below). No long intron-containing bands were found on the agarose gel after amplification of *TRPC2* and *ACTB* and together with the 260/280 absorption ratio and the two steps of genomic DNA elimination during RNA extraction and reverse transcription we exclude any contamination of our samples with genomic DNA.

**Table 1:** **Tissue samples with information about age and sex of animal and season during the time of death**.

**Animal**	**Sex**	**Season**	**Death**	**Age**	**Organ**
Zambo	M	R	2012-04-26	11.9 years	VNO, MOE
Uma	F	R	2013-03-14	7.9 years	VNO, MOE
Vincent	M	NR	2012-10-09	8.3 years	VNO, MOE
Tanja	F	NR	2012-09-20	6.3 years	VNO
Infant	F	-	2013-04-25	10 days	VNO, MOE
Ursina	F	R	2012-03-13	8.9 years	MT

*M, male; F, female; R, Reproductive season; NR, Non-reproductive season; MOE, Main olfactory epithelium; VNO, Vomeronasal organ; MT, Maxilloturbinal. Light conditions during housing were adjusted to the Northern hemisphere*.

### Confirmation of transcribed loci

We developed locus-specific PCR assays to assess the expression of VRs as currently described. From the 107 previously published *V1R* sequences of the gray mouse lemur (Zhang et al., 2010) we excluded 29 loci, because they had no full length sequence (*VN1R Mmur073*) or did not fulfill the following criteria: (1) locus has at least 1% nucleotide differences to other loci (or else one of the two highly similar loci was excluded), (2) primers had not been rejected by the online software Primer3Plus (Untergasser et al., 2007) due to low/high annealing temperature or self assembly using default settings, and (3) the primer pair amplifies at least 500 bp of the target locus. Consequently, we designed locus-specific primer pairs for each of the remaining 78 *V1R* loci. The lowest sequence divergence between two loci for which locus-specific primer pairs were designed was ~3% (=28 bp differences) and amplicons were at least 540 bp long (Table 2). *V1R* loci are named *VN1R Mmur000* to *VN1R Mmur103* (882–1008 bp, Hohenbrink et al., 2012); *V2R* loci are named *VN2R1* (2739 bp) and *VN2R2* (2418 bp) (Hohenbrink et al., 2013).

**Table 2:** **List of locus-specific primer pairs for *V1R* loci with expected PCR product length**.

**Locus name**	**Forward primer**	**Reverse primer**	**Length**
Mmur000	CAGCCAACACCTTTCTCCTG	ATCTATGAGCTTTCTGTTGCATATTC	809 bp
Mmur001	CCAATGTCATTCTGTTCTTCCAT	ACCACAGACGAGAATCAAAAAGTA	676 bp
Mmur004	ACTGGACTTGGCACTGTAGGA	CTGAGAACTGCATAACCAATAGGTAG	750 bp
Mmur007	AGCTGGTCTTAGCCAACAACTTA	GGTTCTGCAGCTGGGTTAG	600 bp
Mmur008	AGTGACAGAATTGTTGGGTCTG	CAAAGGATTGGGTAGCATGAA	793 bp
Mmur010	CGTCCACATACTGGTGCTGT	CAGCATGATGGGACCATAAG	606 bp
Mmur011	GACTGGGAGTAATCTCATCAGGA	CCCTGCCCATCAGCAT	827 bp
Mmur012	CTTCTACAGATGTGGCAATAGGAG	ATTGTATTTCTTCCACAGCAGGT	890 bp
Mmur013	CTTCAGGATCACAGGCCTAAA	ACTGTGGTTGCATATTTTGGAG	765 bp
Mmur020	ATTATCTACTCCTGTGCTTCTCCA	GGGGGTTCTCTTCCTTATCAA	788 bp
Mmur021	CACAATTTTGGGGTTTGTCCTA	GTTGGGTAACATGATGATAAAATTACAA	778 bp
Mmur022	CTTTGTATTGGGGCAATCTCA	AACTTCTCAGTGAATGTGCCAGT	693 bp
Mmur023	GAGTCCTAAGAAAAAGCCCATAGAT	GGCTGAGACTGGCATAACTG	695 bp
Mmur024	CTGGGGAATTTTTCACTCTTGTAT	CGTGGCTCATGAGAACAAAT	764 bp
Mmur025	GATTTTCATGCATCTGACATTG	ACAGTAATTGGATAGAGTTTTGGGTA	630 bp
Mmur027	GTGTAGCCAACTTCTTGGTCATAT	GAAGAAAGGGGCTGACTGC	665 bp
Mmur028	GGAGTAATCTCATCAGGGGAATG	AAATTAGTTTTCTCAACTTCTCAGTGG	865 bp
Mmur030	CACAGCCAATATCTTCCTCCTT	CCGTATGCCACCAACACTAC	687 bp
Mmur031	CTTATCTCTCAGGTTTGTGTTGGTA	ATCAGCAGAAAAGGGCAGAT	782 bp
Mmur033	CATGGGGAACKTCTCTCTTC	TTATCCAGGCAAAGCAAAGC	805 bp
Mmur034	CTCCAGGGATTTGGCAATAA	CCCCAAAGTAGTTGGGACTAGA	886 bp
Mmur036	AATGGGAATGATCTTTCTATCACAA	AGTCGTGGCTCATGAGAATGT	802 bp
Mmur038	GGCCTGTTTGAGTCACTGG	TGCTCATTTTGCAGAGTACTGATA	658 bp
Mmur039	TTTGGCAAGCTGGTATTGGT	CAGGTCCACCCAGTACATGAT	672 bp
Mmur040	GCTGGGGAATTTCCTTCTTC	GCAGAGTGGATTCTGGGTAG	800 bp
Mmur041	GTTTGTCCTTGTCTCTCAGATTTC	CAGCACAAAAGGGCAGAG	790 bp
Mmur043	GCATTGGAGGGAATTTCTTC	CCAGCAGGTAACCAGGTAGAATA	792 bp
Mmur044	GCCTCCAGGGATGTGGA	CAAGCAGCCATTAGTGCAGAC	792 bp
Mmur045	TTTAAGATCATCAAAGGAGCAAT	AGCACAAATGGGCTGAGAAT	802 bp
Mmur046	GCATTGGAGGGAATTTCTTTTTA	GGTGGAACCAATATAGAAGGAGAC	678 bp
Mmur047	CCTATAGATTTGATTTTCATGCATCTT	GGGTTTTTCCGAGGTGTG	623 bp
Mmur048	ATGATTGGGAGTAACCTCATCAC	TAGAATCCCTGCCCATCAGTAT	829 bp
Mmur049	GAGCAATATTCCTTTTTCTAACCA	CCATGTGTCTATCCCTGAAAATC	805 bp
Mmur051	GCCAAGTGGCAATAGGAATC	CATGAGAAGAAAGGGGCTGAT	810 bp
Mmur052	TCTTCCGCACCCTCACA	ACCCAGTACATGAACACAAAGAAG	620 bp
Mmur053	CCTGACTATGGTTGGAATCCTG	ATGAAGCAGAGCCTGGACTT	821 bp
Mmur054	GCATTTTCTTTTGGGACACG	CTGAGAACTGCATAACCAATAGCTAC	702 bp
Mmur057	GAATCCTTGGAAATTTTTCTCTTCTA	GTTTCTAATAAGATTAGGGGATTTTCTG	827 bp
Mmur058	TGTTCAGTACAGTATCCTAAGAACCAC	AAAGAGTGGGAAAACATGAACC	802 bp
Mmur059	CAAGTGAAACAATTTTGGGGATA	ATCAACACAAAAGGGCAGAGT	809 bp
Mmur060	TTGGGGTTTGTCCTCATCTC	CATCAACACAAAAGGGCAGA	800 bp
Mmur061	GGATTGGACTCACAGCCAAC	ACATTCCCCACCAGCGTA	740 bp
Mmur063	TGTGTACTTATATTACATTCAGTCTCACG	AGCACAAAAGGGCAGAGG	721 bp
Mmur064	AACTCATTGCTCTTTGTGTTCTATGTA	ATGACAACATTACAGTAAGTGGATAGAGA	704 bp
Mmur065	TAAAGGAAAATGTGCTGAAGGA	GCCAGAGAAAGCACAGTCG	886 bp
Mmur066	GTCCTGGGGAACACCTTTCT	CTGAGGGTGGGATAACAGGA	748 bp
Mmur067	TGACTGTGGTTGGGGTTGTA	CAATGCCTGGATACACTGGA	807 bp
Mmur068	CACAGATTTGGTTCTCAAGCAG	GTCACGGCCCATGAGAAT	708 bp
Mmur069	ACATATTTCTCATGGGTCCTAAGAT	CCAATCAGTATGAAGGGGCTA	723 bp
Mmur070	GCTAATGCAATGATACTTTGTGCT	ACTTCTCAGTGAATGTGCCAAA	705 bp
Mmur074	ACCTTCCTCCTCCTCTTCCA	TGACACTTCTGTTSCATATTTTG	799 bp
Mmur075	CATATGGGAAGCGGCTCTA	CAAACAGTGGGAAAACATGAACT	786 bp
Mmur076	GGCTGCCTTAGATTTGTCTACAGT	AGATACAAAGGTGCTCACCAGA	690 bp
Mmur077	GATTGGGGTCGGGACTCTA	AGTAAGCATGGAACTCAGCACA	665 bp
Mmur078	TAAATTGGGGCATCACTTTTCTA	GGCATCACTGACAATGAACAG	813 bp
Mmur079	TGCTCACCATGGTGTTTTTC	ACTGTGGCATAGGCACTGA	628 bp
Mmur082	GGGGCATTATCTTTCTTATTCAGT	TAGCCCAGAGAACATACTTGGA	874 bp
Mmur085^a^	TTATCTTTGTCATTCAGACTGGTGT	CCTAGTCCAGCAGGCAAAGT	833 bp
Mmur086	ACAGATTTGGTTCTCAAGCACA	CTTCCACAGCAGGCAGAA	745 bp
Mmur087	GAGAAGTGGCAATAGGAATGATCTA	GCATCGTGTTAGCTTACAGAGTTT	880 bp
Mmur088^a^	TTATCTTTGTCATTCAGACTGGTGT	GACAGAGATGCCCCTCAGAA	740 bp
Mmur094	TCATTCAGACTAGTGTTGGAATCTC	ATGTCGGGAAGCCCAAA	762 bp
Mmur101	TGGAAGCTTTCCTGGATGG	TGGGTTCTGCCGCTGT	540 bp
Mmur103	TTTTATCACTGCACAGAGACTGAATA	CCCAGACCATTCCTAGAGTATTTG	691 bp

a *Mmur085 and 088 share the same forward primer (but reverse primer is specific for each locus)*.

The specificity of the newly designed *V1R* primer pairs was tested by amplifying and sequencing one cDNA sample (VNO tissue, male, reproductive season) and genomic DNA as a positive control. Sequencing genomic DNA enabled confirmation of specificity of each primer pair even if the locus is not expressed in the VNO sample. *V2R* primers were already validated (Hohenbrink et al., 2013). For the positive control genomic DNA was extracted from ear tissue using a DNeasy Tissue Kit (Qiagen) and a REPLI-g WGA kit (Qiagen). All VR fragments were amplified with MyTaq DNA polymerase (Bioline; 25 μl total volume containing 5 μl MyTaq Reaction Buffer, 1 μl of each primer [10 μM stock concentration], 0.1 μl Taq DNA polymerase [5 U/μl] and 1 μl of DNA) with the following PCR conditions: 94°C for 2 min, 40 times (94°C for 30 s, 60°C for 45 s, 72°C for 90 s), 72°C for 5 min. PCR products were sequenced on both strands using BigDye Terminator 3.1 (Applied Biosystems) under standard conditions and run on an Applied Biosystems 3500 capillary sequencing machine. Consensus sequences of single genes were built with SeqMan 5.05 (DNASTAR Inc., Madison, WI, USA). Sequences were aligned and analyzed using MEGA 5 (Tamura et al., 2011). The alignment of the sequenced fragments with the targeted loci showed that 64 of the 78 primer pairs were truly locus-specific. These *V1R* primer pairs (Table 2) and the *V2R* primers were used in subsequent PCRs under the same conditions with all tissue samples and run on 1% agarose gel. The presence and absence of bands was used to confirm or exclude the expression of loci in all tissue samples. All negative PCRs were repeated once to counteract PCR stochasticity and results are presented cumulatively. This method was preferred over next generation sequencing (RNA-Seq) because of the potential difficulty of correctly assembling RNA-Seq data for the large *V1R* gene family, with many closely related sequences.

We were able to design primers for loci of each monophyletic *V1R* cluster known from gray mouse lemurs (Hohenbrink et al., 2012). Four clusters were covered 100% (cluster II, IV, VI, and VII), whereas clusters III (75%) and V (71%) showed high coverage. However, for cluster VIII only one locus (20%) could be sequenced, because the other four loci of the cluster were highly similar and designed primers were not locus-specific. Cluster I (35%; also known as *V1Rstrep*, Yoder et al., 2014) and IX (50%) are the two largest clusters in mouse lemurs and seemed to have evolved by rapid gene duplication. Cluster I was particularly difficult for primer design because of low nucleotide divergence: seven of nine loci that were excluded because of divergence below 1% belonged to this cluster.

## Results

**Figure 1:**
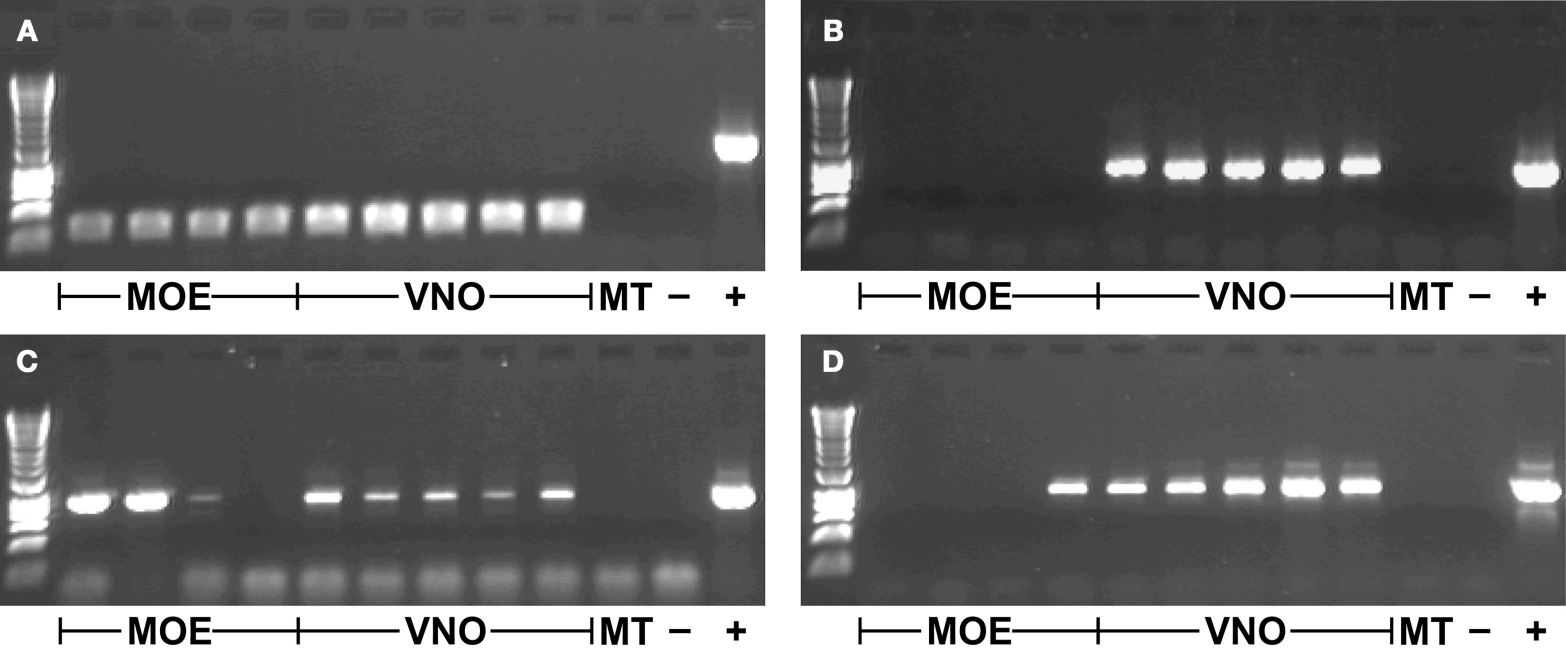
**Gene expression in different tissue samples. (A)** *TRPC2* (~240 bp product); **(B)** *VN1R Mmur078* (~860 bp product); **(C)** *VN1R Mmur028* (~920 bp product); **(D)** *VN1R Mmur034* (~930 bp product); first row contains HyperLadder 1kb (Bioline); MOE, Main olfactory epithelium; VNO, Vomeronasal organ; MT, Maxilloturbinal; -, Blank negative control; +, Positive control of genomic DNA (~1500 bp product for *TRPC2*).

*TRPC2* was expressed in all VNO and MOE tissue samples in the gray mouse lemur (Figure 1A). There was no amplification of *TRPC2* from the negative control of maxilloturbinal tissue, but all samples were positive for *ACTB* (not shown), indicating successful RNA extraction and cDNA synthesis. Each of the 64 *V1R* and the 2 *V2R* loci were expressed in at least one tissue sample (three examples shown in Figures 1B–D). The specific expression patterns of VR loci in VNO and MOE tissues are shown in Table 3. The sample of maxilloturbinal tissue was negative for VR loci.

**Table 3:**
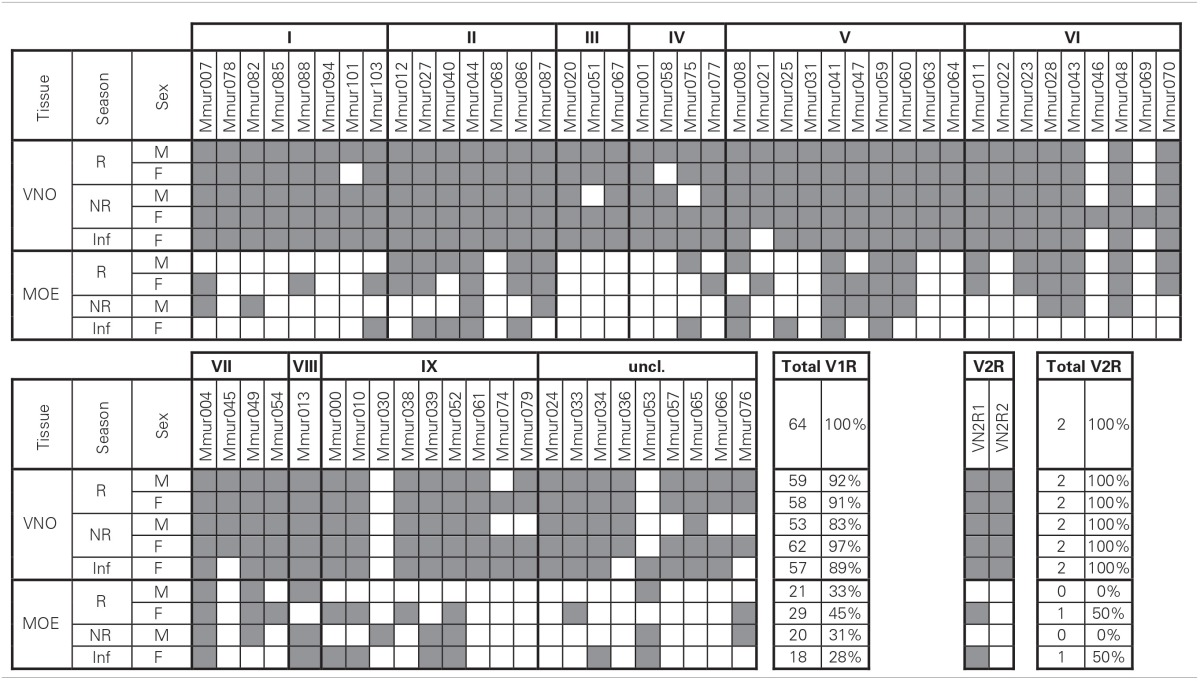
**Expression of vomeronasal receptors in vomeronasal organ (VNO) or main olfactory epithelium (MOE) tissue**.

*Cluster names (I–IX) are given for V1R loci (or uncl., unclustered). Expressed loci are shown in gray. The total number of expressed loci per tissue sample is shown; R, Reproductive season; NR, Non-reproductive season; Inf, Infant; M, Male; F, Female. Primer pairs for the loci VN2R1 Mmur002, 005, 006, 014, 015, 016, 019, 029, 037, 062, 081, 092, 093, and 099 did not work or were not locus-specific. All non-listed loci from VN2R1 Mmur000–106 were excluded due to the three criteria (see Materials and Methods)*.

The proportion of expressed *V1R* loci ranged from 83 to 97% in the VNO and 28–45% in the MOE samples. The number of expressed genes was significantly higher in the VNO than in the MOE (observed vs. expected: χ^2^ > 9.66, *df* = 1, *p* < 0.002 in all four pairwise comparisons). We found no global differences in the number of expressed *V1R*s between the two sexes or the two seasons neither in the VNO nor in the MOE, but one locus (*VN1R Mmur074*) was exclusively expressed in females. There were also no large differences between the adult individuals and the infant. Nevertheless, three loci (*VN1R Mmur021*, *036*, and *045*) were expressed in the VNO samples of all tested adults but were absent in the VNO sample of the infant. Conversely, *VN1R Mmur053* was absent in the VNO tissue of all tested adults but was expressed in the infant.

At least one locus of each of the nine known monophyletic *V1R* clusters was expressed in each of the five VNO tissue samples. Four loci (*VN1R Mmur004*, *041*, *044*, and *059*) were expressed in all nine tissue samples, whereas 25 *V1R* loci (39%) were exclusively expressed in the VNO. Fourteen of these *V1R* loci were expressed in all five tested VNO samples. In total, 48 *V1R* loci (75%) were expressed in all five VNO tissue samples meaning that variation in expression patterns in the VNO was only found in 25% of the loci. More variation existed in the MOE samples. With the exception of three loci (*VN1R Mmur030*, *053*, and *076*) every locus that was expressed in the MOE was also expressed in the corresponding VNO sample of the same individual. The two *V2R* loci were expressed in the VNO of all individuals and the MOE of both females (including the infant) were positive for *VN2R1*.

## Discussion

We have presented the detailed expression patterns of vomeronasal receptors in a primate for the first time. We were able to demonstrate the expression patterns for 64 of 107 *V1R* loci and both known *V2R*s. As expected, the vast majority of the tested *V1R*s and both *V2R*s were expressed in the VNO. However, unexpectedly, a substantial number of VRs (39 *V1R*s and *VN2R2*) were also expressed in the MOE, which, given with *TRPC2* expression in that organ, suggests that these VRs were likely functional. These results reveal a novel organization of the chemosensory system in primates and indicate greater functional overlap between the VNO and MOE than previously recognized.

The majority of the tested VRs (75%) were expressed in all five VNO samples indicating relatively low interindividual variation in the number of expressed VRs. Seasonal differences might have been expected, because some VRs might only be needed during the reproductive season, e.g., to find mating partners or to identify potentially estrous females. However, we did not find a single locus that was solely expressed during the reproductive season in either olfactory organ, although these captive animals show the natural repertoire of seasonal behaviors (e.g., estrus, mating) and seasonal variation in morphology (e.g., testis growth and body mass, Buesching et al., 1998; Radespiel and Zimmermann, 2001; Wrogemann et al., 2001).

Studies on mice showed that expression levels of VRs increased drastically from embryonic to 10-days postnatal age, but only changed marginally within the first 7 months (Zhang et al., 2010). This was in contrast to expression of olfactory receptors in the mouse MOE, where the same study found low expression levels at a lower age with constant increase of expression postnatally until the age of 3–4 months (already adult) with a slight decrease at a later age. This suggests that VRs unlike olfactory receptors need to be highly expressed from early life onwards. This is in concordance with our results in mouse lemurs where the infant showed a high proportion of expressed VRs similar to adult individuals (adulthood in mouse lemurs reached after less than 1 year, Glatston, 1979), although no information about actual expression levels are available. Additionally, vomeronasal neuroepithelium is already present at early prenatal stages (Garrett et al., 2013) and numerous neurons reactive to Olfactory Marker Protein—a marker of terminally differentiated vomeronasal and olfactory neurons—were found in the VNO and MOE sensory epithelium of a 1-day old mouse lemur indicating olfactory functionality at this age (Smith et al., 2007). Infant mouse lemurs open their eyes at the postnatal age of 4–6 days, acoustically communicate with their mothers (Glatston, 1979; Scheumann et al., 2007b), and start to leave the nest by the age of ~21 days (Lutermann, 2001). Our results imply the potential for the use of olfactory signals between infants and nest mates (including mothers and siblings) or the early perception of predator cues.

In our study one male was almost 12 years old when he died. Wild mouse lemurs suffer from high predation (Scheumann et al., 2007a) and a near-complete population turnover has been reported after 5 years (Kappeler and Rasolarison, 2003; and see Lutermann et al., 2006). In captivity mouse lemurs in a normal photoperiodic regime are considered to be aged beyond the age of 7.5 years (Zimmermann and Radespiel, unpublished results) and can show signs of senescence. Following this definition three of four tested adult individuals were aged. However, aged mouse lemurs did not perform significantly worse than young animals in olfactory discrimination tasks, and only a minority of aged individuals showed altered behavior during an olfactory reversal learning task (Joly et al., 2006). Given the high proportion of expressed genes in all our individuals, we do not predict any decreased functionality of the olfactory organs due to senescence, although deteriorating central nervous processing would be possible in analogy to the hearing system (Schopf et al., 2014). Another explanation for an ongoing importance of the olfactory sense in aged animals could be the need to compensate an age-dependent decline of other senses (e.g., vision) with olfaction. However, although age-related visual impairments like cataracts and blindness have been described in captive gray mouse lemurs (Beltran et al., 2007), the individuals in our study were not visually impaired (Dubicanac, personal communication). A study on mice showed evidence of seven age-dependent expression profiles for VRs (Zhang et al., 2010), but testing only one infant and older adults did not allow to detect such profiles in our study. Mouse lemurs were not sacrificed for the purpose of this study and available samples were therefore limited. Animals would have to be euthanized systematically at different ages and in large numbers to collect information about age-dependent expression which cannot be supported for these primates from an ethical point of view. The sporadic availability of samples makes it also difficult to do *in situ*-hybridization in mouse lemurs on a larger scale.

We only found sex differences in expression in the VNO at a single locus. We assume that a large number of VRs are used to detect predator cues or signature mixtures to identify individuals and these types of information are equally important for both sexes, e.g., to minimize the predation risk or avoid inbreeding. Pheromones used for intraspecific communication should be equally relevant for males and females in most cases. Touhara and Vosshall (2009) assumed that male and female mice have the same set of pheromone receptors and that both sexes might show behavioral differences because of sex-specific neural circuits in the brain. Sex-specific signal transmission has been reported in mice where the same ligand and *V2R* receptor pair induces different behaviors in males and females (Haga et al., 2010; but also Halem et al., 1999). The VNO was also reported to be larger in male rats than in females (Segovia and Guillamón, 1982), but no sex differences in VR expression are known. It has to be mentioned, though, that differences found between the VNO samples in our study could also indicate individual differences. *V1R*s show monoallelic expression (Rodriguez et al., 1999; Roppolo et al., 2007) and our reported high number of expressed and most likely functional loci highlights the important role of the vomeronasal system for the sensory ecology of mouse lemurs. The function of each VR, however, is still unclear and has to be analyzed in further studies.

In the present study a large proportion of VRs (59%) as well as *TRPC2* were also expressed in at least one MOE sample. This result was specific to MOE and VNO, since no VR or *TRPC2* expression were detected in adjacent maxilloturbinal tissue. *TRPC2* is essential for the functionality of the VNO, and is required for signal transduction of both *V1R*s and *V2R*s (Liman et al., 1999). Male mutant mice lacking *TRPC2* did not attack intruding males and indiscriminately mounted males and females (Leypold et al., 2002; Stowers et al., 2002). In our study both the VNO and MOE of mouse lemurs did express *TRPC2* indicating functionality of the expressed VR genes in these chemosensory organs. In contrast, whereas in mice expression of *TRPC2* in the VNO is similarly strong, only “weak” expression of *TRPC2* has been so far reported in the MOE (Liman et al., 1999). The apparently strong expression of *TRPC2* in the mouse lemur MOE was therefore unexpected, but in concordance with the expression of a large number of VRs in this organ. However, a quantitative comparison between *TRPC2* expression in mice and mouse lemurs is not yet available. Expression of single *V1R*s in the MOE was reported in goats, mice and humans (Rodriguez et al., 2000; Wakabayashi et al., 2002; Karunadasa et al., 2006; Ohara et al., 2009; Pascarella et al., 2014). It was also shown in mice that 2-heptanone, a pheromone that binds the *V1rb2* (Boschat et al., 2002), elicited strong signals in both the main and the accessory olfactory bulb (Xu et al., 2005), the brain structures that receive projections from the MOE or VNO, respectively (see Munger et al., 2009). Here we have found expression of far more VRs in the MOE of mouse lemurs than previously described in any primate, including many loci expressed both in the MOE and VNO. The results were strengthened by the expression of *TRPC2* in the MOE. Three main hypotheses may explain the involvement of the MOE in VR-mediated chemosensory pathways: (1) different ligand sensitivities in the two organs, (2) better coordination between the two organs, or (3) different downstream neural pathways of the two organs.

The first hypothesis incorporates structural differences between MOE and VNO that facilitate the intake of volatile or non-volatile molecules, respectively. This argument is supported by the finding that in mouse lemurs the volatile phase of urine activates the MOE but not the VNO, which is only stimulated by urine in the liquid phase (Schilling et al., 1990). As the VNO lumen is filled with fluid that can be set in motion by vomeronasal pumps in the organ (Meredith et al., 1980; Meredith, 1994), volatile molecules reach the vomeronasal sensory epithelium less easily than non-volatile. Therefore, pheromones or other ligands bound by VRs being more or less volatile might be better perceived by one or the other olfactory organ. However, recent studies reject this classical view as both organs can perceive volatile as well as non-volatile pheromones (reviewed in Zufall and Leinders-Zufall, 2007). Moreover, it would remain unclear, why the same VRs are expressed in both organs.

The other two hypotheses may explain the simultaneous expression: Ohara et al. (2009) suggested that *V1R*s expressed in the MOE of goats might first detect pheromones that induce the flehmen response (animal raises the head and curls back the upper lip to facilitate the inflow of molecules to the VNO). According to the authors the pheromones are then quantitatively analyzed with the *V1R*s in the VNO and levels of airborne pheromones may be too subtle to be detected without the coordination of both olfactory organs.

However, as flehmen is not described in mouse lemurs (but found in ring-tailed lemurs, Bailey, 1978), we present a third hypothesis: the expression of VRs in the MOE may be explained by the use of different neural pathways in both olfactory systems processing the signals in various brain regions (Mestre et al., 1992; Meisami and Bhatnagar, 1998; Meredith, 1998 and see review in Dulac and Wagner, 2006; Touhara and Vosshall, 2009). The MOE projects to the main olfactory bulb that after accessing paleocortical nuclei is connected to higher brain centers (Lledo et al., 2005) allowing adaptive responses based on experience. In contrast, the VNO bypasses cortical structures and projects directly to nuclei of the limbic system which mediates innate responses (Meisami and Bhatnagar, 1998; von Campenhausen and Mori, 2000; Dulac and Wagner, 2006). Given the potential complexity in olfactory signal composition and transmission in a small nocturnal solitary forager such as the mouse lemur and the fundamental structural separation of the two olfactory organs including different neural pathways, it is likely that evolution may have favored some degree of redundancy in the involved cells, receptors and structures to improve sensory abilities. For example, the availability of VR receptors in the MOE would allow mouse lemurs to better process the various olfactory signals that are produced and deposited in several species-specific marking behaviors (e.g., anogenital marking, head rubbing, urine washing, Glatston, 1979; Buesching et al., 1998; Braune et al., 2005).

The expression results showed a higher variation of expressed VRs in the MOE than in the VNO. A potential explanation for this finding could be spatial competition of VR- and olfactory receptor expressing neurons in the MOE. The more than 300 different cells that express olfactory receptors (Matsui et al., 2010) dilute the concentration of VRs making them harder to be detected with our approach. Such spatial limitations could also have favored rather temporal expression of single VRs (possibly based on experience) that could lead to more plasticity in the neuronal circuits. The high variation of expressed VRs in the MOE could also reflect a high variability in the gene regulation of these primates.

Future studies are needed to identify the full genomic repertoire of *V1R*s and *V2R*s based on the complete genome of mouse lemurs which has been sequenced at >160× coverage by Rogers and colleagues at the Baylor College of Medicine Human Genome Sequencing Center and is currently in the assembly and annotation phase (Yoder, personal communication). We hypothesize that the simultaneous expression of a large number of the same VRs in MOE and VNO—which has never been shown in any primate species before—has evolved in mouse lemurs to adequately process a variety of complex olfactory signals, as separate neural pathways of both olfactory systems project to different brain regions performing special functions. Our results indicate a further blurring in the long presumed functional distinction between the VNO and MOE, following on from demonstration of pheromone detection in the MOE of some species, in some cases by olfactory receptors (Hudson and Distel, 1986; Swann et al., 2001; Charra et al., 2012). More emphasis is needed on comparative adaptive function of these VRs in the MOE and VNO of mouse lemurs and other species with large VR repertoires and highly developed olfactory sense.

## Author contributions

The conception and design of the study was done by Philipp Hohenbrink, Nicholas I. Mundy and Ute Radespiel. Material was provided by Elke Zimmermann. The data were acquired by Philipp Hohenbrink and Silke Dempewolf and analyzed by Philipp Hohenbrink. The interpretation of the data was done by Philipp Hohenbrink, Nicholas I. Mundy and Ute Radespiel. The article was drafted by Philipp Hohenbrink and critically revised by all authors. All authors approved the publication of the final version.

### Conflict of interest statement

The authors declare that the research was conducted in the absence of any commercial or financial relationships that could be construed as a potential conflict of interest.

## Abbreviations

MOE: Main olfactory epithelium
TRPC2: Transient receptor potential channel type C2
VNO: Vomeronasal organ
V1R: Vomeronasal receptor type 1
V2R: Vomeronasal receptor type 2
VR: Vomeronasal receptor.
